# A retrospective study of severity-dependent HPG Axis suppression by glucocorticoids in Chinese women with Cushing syndrome

**DOI:** 10.3389/fendo.2026.1681387

**Published:** 2026-06-29

**Authors:** Anting Yu, Xuan Liu, Yiyu Chen, Shuo Li, Ming Liu

**Affiliations:** Department of Endocrinology and Metabolism, Tianjin Medical University General Hospital, Tianjin, China

**Keywords:** Cushing syndrome, ectopic ACTH syndrome, HPG axis, mass spectrometry, serum steroids

## Abstract

**Background:**

Cushing syndrome (CS) is a severe endocrine disorder caused by prolonged exposure to glucocorticoid excess. Among its diverse complications, reproductive and sexual disorders arehighly prevalent, impacting patients’ quality of life. Despite this clinical significance, clinicalstudies specifically investigating the hypothalamic-pituitary-gonadal (HPG) axis status in femalepatients with CS remain insufficient, highlighting the urgent need for thorough evaluation.

**Method:**

This retrospective cross-sectional study evaluated the HPG axis status in 137 women diagnosed with CS between 2007 and May 2024, comprising adrenal CS, Cushing’s disease (CD) and ectopic adrenocorticotropic hormone (ACTH) syndrome (EAS), with reproductive hormone profiles compared across the three subtypes. Linear regression analysis was employed to assess the association between reproductive hormone levels and the severity of hypercortisolism. In a subgroup of 45 women with available steroid profiles quantified using liquid chromatography-tandem mass spectrometry (LC-MS/MS), plasma androgens were evaluated through receiver operating characteristic (ROC) curve analysis to determine their diagnostic power. Additionally, the relative susceptibility of the thyroid axis and the gonadal axis to hypercortisolism was comparatively examined.

**Results:**

Among the three CS subtypes, female patients with EAS exhibited markedly elevated ACTH, serum cortisol, and 24-hour urinary free cortisol (24h-UFC), alongside markedly elevated testosterone and reduced luteinizing hormone (LH) and follicle-stimulating hormone (FSH) levels. Linear regression analysis revealed significant positive associations between serum cortisol with testosterone, whereas inverse correlations were observed with LH and FSH, particularly in postmenopausal women. Furthermore, plasma androgens, including testosterone, androstenedione (A2), dehydroepiandrosterone (DHEA), and dehydroepiandrosterone sulfate (DHEAS), demonstrated superior diagnostic power in discriminating CD from adrenal CS. Additional comparative analyses between the thyroid axis and the gonadal axis indicated that the latter exhibited greater susceptibility to hypercortisolism.

**Conclusion:**

Our findings demonstrate that a severity-dependent glucocorticoid suppression of HPG axis function across CS subtypes, with the most pronounced effects observed in EAS, which may facilitate clinical identification.

## Introduction

Cushing syndrome (CS) is a chronic and systemic endocrine disease caused by long-term exposure to excessive glucocorticoids, leads to a myriad of clinical manifestations and complications (1). CS is traditionally categorized into two subgroups: ACTH-dependent CS, accounting for 70-80% of CS, and ACTH-independent CS (AI-CS) (2). ACTH-dependent CS is most commonly caused by Cushing’s disease (CD) (80-90%), while ectopic ACTH secretion (EAS) is less frequent (10-20%) and poses diagnostic challenges (3–5). ACTH-independent CS is primarily caused by unilateral adrenal adenomas (70-80%), while adrenal carcinomas almost covered the remaining(20–30%) (2).

The clinical complications of CS primarily include systematic arterial hypertension, glucose and lipid metabolism disorders, musculoskeletal disorders, neuropsychiatric disorders, dermatological manifestations, and impairment of reproductive and sexual function. Reproductive and sexual disorders, primarily hypogonadism in men and menstrual irregularities in women, are common in patients with CS (6, 7). CS predominantly affects women, frequently presenting with menstrual abnormalities, acne, hirsutism, and polycystic ovaries (8). However, there are only handful clinical studies focused on how CS manifests in women. One recent study demonstrated differences in etiologies and clinical presentation across age groups, indicating women ≥65 years presented with less characteristic features (9). Another very old study involving only 45 premenopausal women with CD revealed that menstrual disturbances are closely associated with hypercortisolemia rather than androgens (10). Other existing studies are largely limited to males (11, 12), leaving questions unanswered in women. Although it’s well known that glucocorticoid is crucial to the establishment and maintenance of reproductive function, producing a spectrum of effects on both male and female fertility, its molecular mechanisms still remain partly unclear (13–15).

Women exhibit greater glucocorticoid responsiveness and also a higher prevalence of CS (16), yet, there is a notable lack of research examining the clinical status of the HPG axis in women with CS, especially regarding menopausal status differences. Hormone levels fluctuate markedly across the menstrual cycle, necessitating menstrual phase stratification in clinical studies (17). To our knowledge, only one study has stratified women with CS by menopausal status, reporting 13 premenopausal and 16 postmenopausal patients. But the sample size was limited and the predominance of CD in both groups (n=8 each) limit broader inferences (18). This gap is largely attributed to the complexity of the menstrual cycle and the limited sample sizes in studies, given CS is a relatively rare disease (19).

Therefore, the aim of this retrospective study is to evaluate the clinical status of the HPG axis in women with CS, with a focus on the influence of different etiologies and varying intensity of hypercortisolism in both pre- and postmenopausal women.

## Materials and methods

### Setting

This was a retrospective cross-sectional study at the department of Endocrinology and Metabolism at Tianjin Medical University General Hospital. All coded medical records of women patients with CS assessed in routine care between 2007 and May 2024 were analyzed. All blood samples were collected at the time of initial diagnosis, prior to any therapeutic intervention.

### Ethical approval

The studies involving human participants were reviewed and approved by the Institutional Review Board of Tianjin Medical University General Hospital. The requirement for written informed consent was waived by the ethics committee because all patient data were retrospectively extracted from the hospital’s electronic medical records and fully anonymized prior to analysis.

### Clinical evaluation

**Figure 1:**
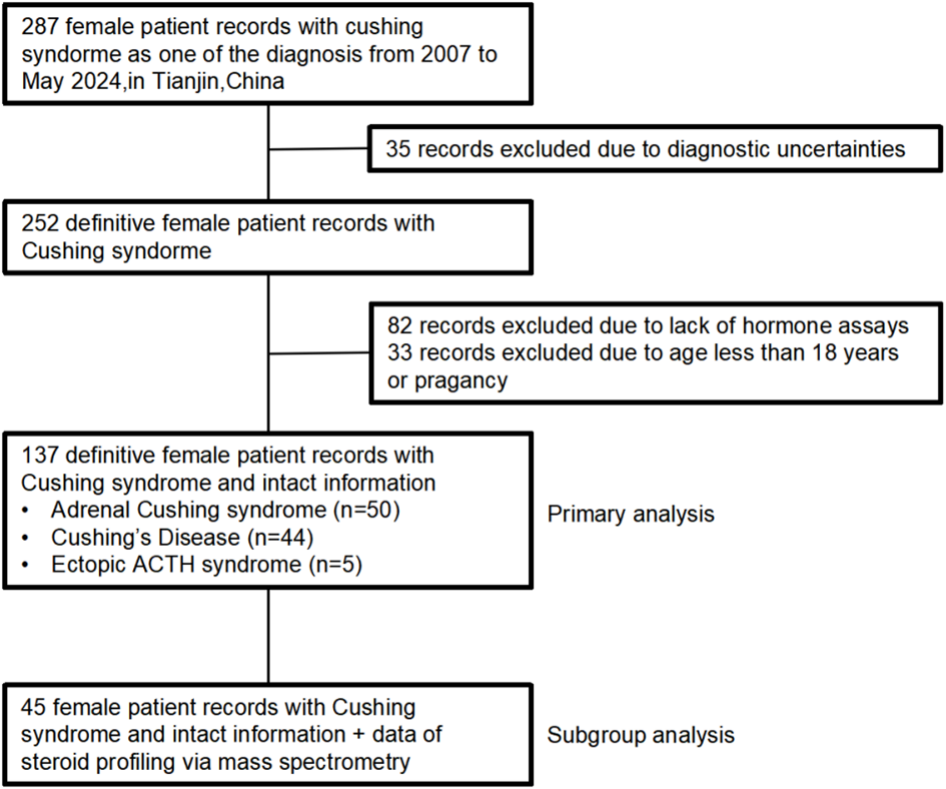
Flow chart of the selection process and the design of the study. 137 female patients meet the criterion for primary analysis, among them 45 underwent mass spectrometry for steroid profiling; ACTH, adrenocorticotropic hormone.

All patients were hospitalized at Tianjin Medical University General Hospital to undergo a full diagnostic work-up. The selection process is shown in flow chart (**Figure 1**). Exclusion criteria were: (1)lack of data on sexual hormone assays; (2)subclinical CS, iatrogenic CS, or other diagnostic uncertainties; (3)patients under 18 years of age or pregnancy.

The diagnosis of Cushing syndrome was based on clinical features and at least 2 of the following: (1) 24-h urinary free cortisol (24h-UFC) excretion (>0.109 mg/24h) in at least 2 samples; (2) 1-mg overnight dexamethasone suppression test (08:00 h plasma cortisol>50 nmol/L) (20); (3) 48 h, 2 mg/d dexamethasone suppression test urinary free cortisol>27 nmol/24 h (21).The 1-mg overnight dexamethasone suppression test was performed as a screening test for CS, based on the principle that exogenous glucocorticoid administration suppresses the hypothalamic-pituitary-adrenal (HPA) axis via negative feedback in healthy individuals, whereas this suppression is impaired in patients with CS due to autonomous cortisol secretion (20). The 48 h, 2 mg/d dexamethasone suppression test was used to aid in the differential diagnosis between Cushing’s disease and ectopic ACTH syndrome. The rationale is that ACTH-secreting pituitary adenomas retain partial glucocorticoid sensitivity and thus show cortisol suppression at high dexamethasone doses, whereas ectopic ACTH-secreting tumors are typically resistant (21).

The majority of the diagnoses of adrenal Cushing syndrome were histologically confirmed. The diagnosis of Cushing’s disease was established if pathological examination of the pituitary surgical specimen confirmed the diagnosis, or if the condition resolved after resection or irradiation of the pituitary gland. The diagnosis of an ectopic cause of ACTH secretion was confirmed by histological examination of surgical or biopsy specimen. Nonetheless, not all sources of ACTH excess were confirmed, in this case, presumed diagnosis was extracted from diagnostic code at discharge. The diagnoses were made at the discretion of the attending physician, based on clinical presentation, results of high-dose dexamethasone test, pituitary MRI, chest and abdominal CT.

### Hormone assays

Preoperative blood samples were obtained in the morning between 06:00 to 08:00 from an 8h fasting blood draw to minimize potential confounding introduced by circadian rhythmicity and food intake. Plasma ACTH, cortisol and 24-h excretion of urinary free cortisol were measured using chemiluminescent enzyme immunoassay (Siemens Healthcare Diagnostics Inc., Erlangen, Germany). The reproductive hormones (FSH, LH, estradiol, testosterone) were measured at the early time of primary diagnosis, and determined by chemiluminescence as well.

Only 45 women with CS were available to measure the plasma steroids by LC-MS/MS, and the steroid profile includes the following: progesterone, 17-hydroxyprogesterone, DHEA, DHEAS, androstenedione (A2), testosterone, 11-deoxycorticosterone, 11-deoxycortisol, cortisol, corticosterone and cortisone. After appropriate sample preparation, the blood was centrifuged at 14,000g for 10 minutes. A 100 µL aliquot of the supernatant was then analyzed using a Jasper™ high-performance liquid chromatography system coupled with an ABSCIEX Triple Quad™ 4500 MD mass spectrometer, equipped with a heated nebulizer ionization source operating in positive ion mode. Quantification of the data was performed using MultiQuant™ MD 3.0.3 software. Of note, testosterone levels measured by LC-MS/MS are generally lower than chemiluminescence immunoassay due to higher specificity and absence of cross-reactivity (22). Therefore, values from the two methods should not be directly compared.

Thyroid function tests were extracted from the medical records as part of the comprehensive endocrine evaluation routinely performed in all patients with suspected CS at our center. Because chronic hypercortisolism is known to suppress the hypothalamic-pituitary-thyroid (HPT) axis, these data are reported to provide a complete baseline endocrine profile of the study population.

### Statistical analysis

Statistical analysis was performed using GraphPad Prism version 9.0, IBM SPSS Statistics version 25 and R (a language and enviroment for statistical computing). Standard descriptive analyses were used to summarize the study variables. Shapiro’s test was applied to test for normality. For quantitative variables,comparisons between controls and cases at baseline were performed by unpaired Student’s t-tests (or Wilcoxon tests if the distribution of the variable was not log-normal). Comparison between the three subgroups of CS was conducted by ordinary one-way ANOVA in case of homoscedasticity or Kruskal-Wallis instead. Tukey’s test (respectively Games-Howell’s test) was used for the a posteriori multiple comparisons. Simple linear regression was used to assess the unadjusted association between indices of hypercortisolism (serum cortisol, ACTH, 24h-UFC) and reproductive hormone levels. Given the known differences in reproductive hormone regulation between premenopausal and postmenopausal women (23), regression analyses were performed separately by menopausal status. The receiver operating characteristic (ROC) curve analysis was performed to test the usefulness of plasma androgens as markers for differentiating between adrenal CS and CD. A total of 45 patients (adrenal CS, n=24; CD, n=21) were included in the ROC analysis. The area under the curve (AUC) with 95% confidence intervals (CI) was calculated for each androgen marker. The optimal cutoff values were determined using the Youden index (sensitivity + specificity -1). A p-value <0.05 was considered statistically significant.

## Results

### Demographic and clinicopathological characteristics

The study cohort was derived from 287 female patients evaluated for suspected CS at Tianjin Medical University General Hospital between 2007 and May 2024. Following rigorous biochemical confirmation as detailed in methods (**Figure 1**), we excluded 150 cases comprising subclinical CS, iatrogenic CS, and patients lacking sexual hormone assays, under 18 years of age, or pregnancy. The final analytical cohort comprised 137 confirmed CS cases, including 63 adrenal adenomas (45.9%), 57 Cushing’s disease cases (41.6%), and 17 ectopic ACTH syndrome (EAS) cases (12.4%) for primary analysis. The EAS cases demonstrated diverse neuroendocrine origins confirmed through histopathological examination (5): thymic neuroendocrine tumors (NETs, n=5), gastrointestinal NETs (n=3), pheochromocytomas (n=2), pulmonary NETs (n=1), pancreatic NET (n=1), small cell lung carcinoma (n=1), and renal clear cell carcinoma (n=1).

### HPG axis in women with Cushing syndrome

The baseline characteristics and hormonal profiles of the study cohort are summarized in **Table 1**. No significant differences in body mass index (BMI) were observed among the three subgroups, while the mean age of patients with EAS was higher than that of the other two subgroups, consistent with previous reports (4). As expected, the three subgroups of women with CS exhibited markedly distinct levels of ACTH (AI-CS < CD < EAS). However, the severity of hypercortisolism, as measured by serum cortisol and 24h-UFC, was significantly elevated only in the EAS subgroup (P < 0.001), with no significant differences were observed between adrenal CS and CD.

**Table 1:** Characteristics of 137 women with Cushing syndrome.

Variables	Adrenal Cushing syndrome (n=63)	Cushing’s disease (n=57)	Ectopic ACTH syndrome (n=17)	P-value
Age (years)	40.11 ± 12.60	39.95 ± 11.09	55.65 ± 16.15	**<0.001**
Body mass index (BMI)(kg/m2)	26.15 ± 4.19	26.92 ± 4.83	27.09 ± 5.31	0.587
ACTH (pg/ml)	5.00(5.00,6.79)	66.40(46.55,114.00)	225.00(123.00,481.00)	**<0.001**
Serum cortisol (ng/ml)	30.85 ± 8.75	33.07 ± 11.53	51.04 ± 17.01	**<0.001**
Urinary-free cortisol (mg/24 h)	450.00(193.50,650.00)	412.20(174.65,592.80)	1424.50(945.00,2081.00)	**<0.001**
FSH (IU/L)	4.54(3.00,7.48)	5.18(3.58,8.25)	1.70(0.79,3.32)	**<0.001**
LH (IU/L)	3.29(1.32,6.21)	2.58(1.06,6.92)	0.16(0.07,0.39)	**<0.001**
E2 (pg/ml)	34.00(15.00,91.00)	32.86(14.71,54.00)	22.80(14.50,31.67)	0.365
T (ng/dl)	24.15(17.79,40.23)	52.00(38.77,72.78)	105.50(65.26, 199.80)	**<0.001**
FSH: LH ratio	2.01(1.20,3.87)	2.62(1.64,4.64)	10.83(3.41,20.78)	**<0.001**
FT3 (pmol/L)	3.21 ± 0.66	3.29 ± 0.81	2.64 ± 0.72	**0.005**
FT4 (pmol/L)	11.274 ± 1.778	11.556 ± 2.470	11.86 ± 3.54	0.613
TSH (uIU/mL)	0.69(0.53,1.22)	0.62(0.36,1.14)	0.49(0.23,0.91)	0.097
FT3:FT4 ratio	0.29(0.25,0.31)	0.28(0.25,0.32)	0.23(0.18,0.27)	**<0.001**

Statistically significant results (P<0.05) were highlighted in bold. Comparison between the three groups was performed using one-way ANOVA or Kruskal-Wallis test if the distribution of the respective variable was not log-normal in any of the groups. ACTH, adrenocorticotrophic hormone; FSH, Follicle-stimulating hormone; LH, Luteinizing hormone; E2, estradiol; T, testosterone; TSH, thyroid-stimulating hormone.

**Figure 2:**
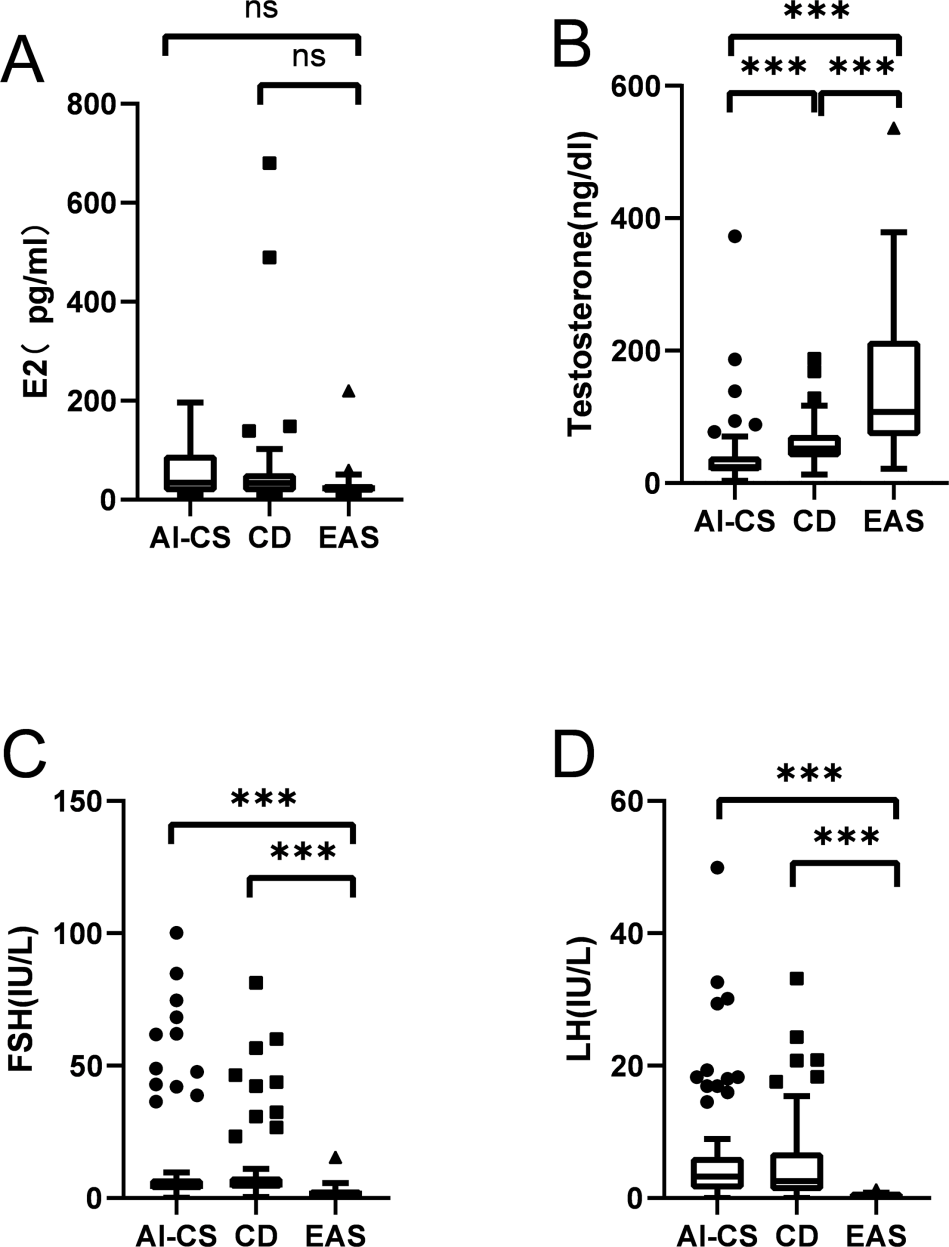
Box-and-Whisker Plots of reproductive hormones. Estradiol, testosterone, LH, and FSH values are displayed in **(A–D)**, respectively. All comparison between the three subgroups are shown by brackets, asterisks or text. The observed outlier distribution patterns align with natural biological variation. E2, estradiol; FSH, Follicle-stimulating hormone; LH, Luteinizing hormone; AI-CS, ACTH-independent Cushing syndrome; CD, Cushing’s disease; EAS, ectopic ACTH syndrome. ***P<0.001; ns, not significant.

Comparative analyses of reproductive hormones among the CS subtypes are detailed in **Figure 2**. Serum estradiol levels did not significantly differ between adrenal CS, CD, and EAS, remaining unaffected by the degree of glucocorticoid excess in this patient cohort. In contrast, testosterone levels were significantly elevated in women with EAS and CD compared to adrenal CS (P < 0.001), with the highest levels observed in EAS (P < 0.001) (**Figures 2A, B**). Circulating LH and FSH levels were notably reduced in women with EAS, differing considerably compared to the other two subgroups, while no meaningful distinctions were observed between adrenal CS and CD (**Figures 2C, D**).

**Figure 3:**
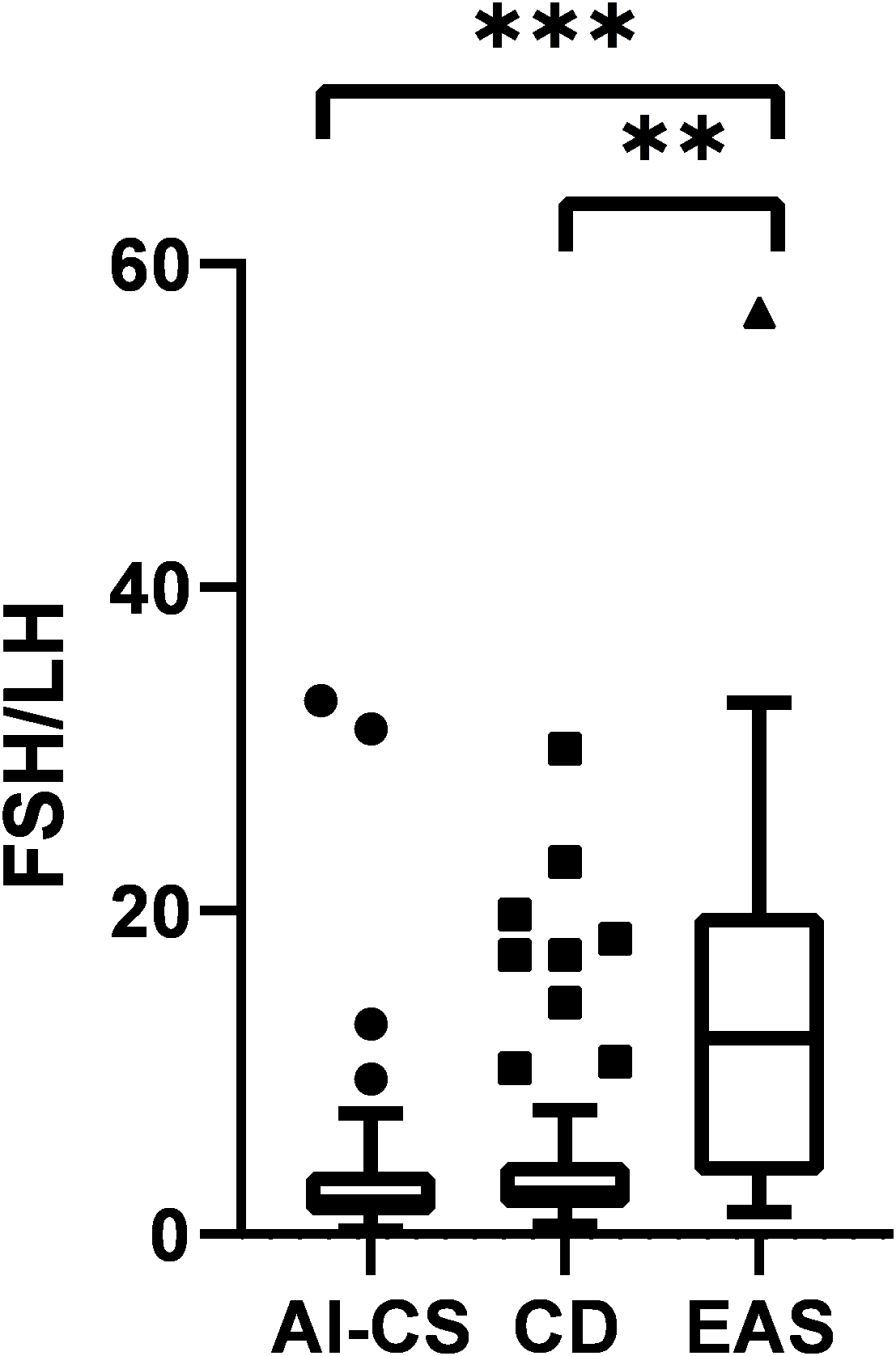
Box-and-Whisker Plots of FSH: LH ratios. All comparison between the three subgroups are shown by brackets,asterisks or text. FSH, Follicle-stimulating hormone; LH, Luteinizing hormone; AI-CS, ACTH-independent Cushing syndrome; CD, Cushing’s disease; EAS, ectopic ACTH syndrome. ***P<0.001; **P<0.01.

Additionally, as a reported predictor of decreased ovarian reserve and ovarian response to exogenous gonadotropin (24, 25), the FSH: LH ratio was calculated and compared across the subgroups.Women with EAS exhibited a significantly higher FSH: LH ratio compared to both adrenal CS (P < 0.001) and CD (P = 0.002) (**Figure 3**).

### Linear regression between hypercortisolism severity and reproductive hormones in premenopausal and postmenopausal women

**Figure 4:**
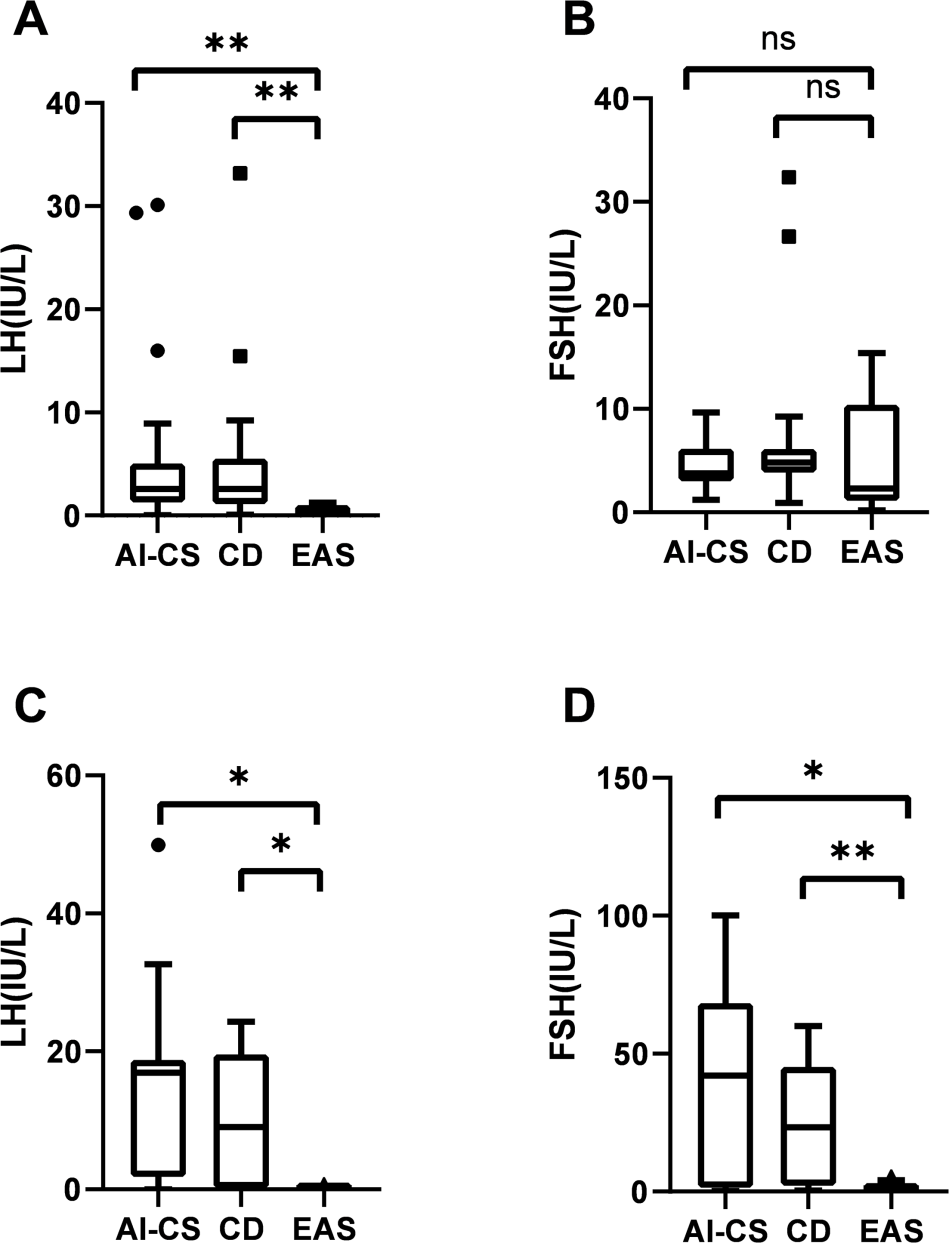
Box-and-Whisker Plots of LH and FSH in women separated by menstruation. **(A, B)**, premenopausal women with CS were included. **(C, D)**, postmenopausal women with CS were included. All comparison are shown by brackets,asterisks or text. FSH, Follicle-stimulating hormone; LH, Luteinizing hormone; AI-CS, ACTH-independent Cushing syndrome; CD, Cushing’s disease; EAS, ectopic ACTH syndrome. **P<0.01; *P<0.05; ns, not significant.

Given the well-established periodicity of reproductive hormones and their central role in menstrual regulation (23), the study cohort was stratified by menopausal status to minimize cyclical fluctuations, as detailed in **Table 2**. After the stratification, the three subgroups still exhibited markedly increased levels of ACTH, with serum cortisol and 24h-UFC significantly higher only in women with EAS. Notably, the suppression of HPG axis was more pronounced in postmenopausal women with EAS, reflected by lower LH and FSH levels in postmenopausal subgroups (**Figure 4**). The ACTH and testosterone levels remained higher in CD than in adrenal CS in both premenopausal and postmenopausal subgroups.

**Table 2:** Characteristics of women with Cushing syndrome separated by menstruation.

Premenopause (n=99)				
Variables	Adrenal Cushing’s syndrome (n=50)	Cushing’s disease (n=44)	Ectopic ACTH syndrome (n=5)	P-value
Age (years)	34.98 ± 7.99	35.27 ± 7.53	34.20 ± 9.91	0.953
Body mass index (BMI)(kg/m2)	26.05 ± 4.31	27.88 ± 4.60	30.23 ± 8.59	0.149
Serum cortisol (random)(ng/ml)	31.22 ± 8.76	32.62 ± 11.46	52.75 ± 3.78	**<0.001**
ACTH (pg/ml)	5.00(5.00,5.75)	65.55(46.28,108.95)	741.00(123.00,1083.00)	**<0.001**
Urinary-free cortisol(mg/24 h)	490.00(259.75,701.35)	420.70(190.40,584.10)	2000.00(1225.00,2306.00)	**0.001**
FSH(IU/L)	4.08(3.07,6.43)	4.84(3.90,6.14)	2.300(1.10,10.37)	0.392
LH(IU/L)	2.59(1.29,5.04)	2.57(1.13,5.51)	0.40(0.35,1.00)	**0.009**
E2(pg/ml)	47.00(21.54,97.65)	36.00(22.50,62.06)	28.72(26.29,135.50)	0.620
T(ng/dl)	24.42(16.71,41.21)	52.48(41.48,71.40)	244.20(139.95, 457.46)	**<0.001**
FT3 (pmol/L)	3.22 ± 0.66	3.39 ± 0.81	2.86 ± 0.91	0.247
FT4 (pmol/L)	11.245 ± 1.802	11.715 ± 2.496	11.98 ± 3.17	0.518
TSH (uIU/mL)	0.93(0.11,4.07)	1.05(0.01,4.29)	0.30(0.01,0.49)	0.169
Postmenopause (n=38)				
Variables	Adrenal Cushing’s syndrome (n=13)	Cushing’s disease (n=13)	ectopic ACTH syndrome (n=12)	P-value
Age (years)	59.85 ± 5.24	55.77 ± 4.34	64.58 ± 6.88	**0.001**
Body mass index (kg/m2)	26.55 ± 3.85	23.67 ± 4.24	26.79 ± 4.51	0.127
Serum cortisol (random)(ng/ml)	29.41 ± 8.94	33.49 ± 11.68	50.33 ± 20.34	**0.002**
ACTH (pg/ml)	5.00(5.00,9.32)	66.40(40.25,128.00)	220.50(83.45,328.00)	**<0.001**
Urinary-free cortisol (mg/24 h)	299.00(147.75,503.10)	233.80(130.53,721.70)	1212.50(785.00,2012.50)	**0.001**
FSH(IU/L)	42.04(1.38,68.35)	23.33(2.14,45.13)	1.230(0.78,2.44)	**0.012**
LH(IU/L)	16.93(1.82,18.79)	9.03(0.18,19.56)	0.150(0.07,0.25)	**0.021**
E2(pg/ml)	11.00(10.00,16.50)	12.690(10.00,28.50)	16.00(13.10,28.46)	0.267
T(ng/dl)	24.15(20.64,39.13)	47.70(33.17,73.56)	85.26(55.85,114.44)	**0.008**
FT3 (pmol/L)	3.16 ± 0.65	2.97 ± 0.71	2.51 ± 0.66	0.071
FT4 (pmol/L)	11.386 ± 1.746	11.065 ± 2.311	11.817 ± 4.007	0.797
TSH (uIU/mL)	0.81(0.3,1.46)	1.11(0.03,6.79)	0.66(0.04,1.51)	0.595

Statistically significant results (P<0.05) were highlighted in bold. Comparison between the three groups was performed using one-way ANOVA or Kruskal-Wallis test if the distribution of the respective variable was not log-normal in any of the groups. ACTH, adrenocorticotrophic hormone; FSH, Follicle-stimulating hormone; LH, Luteinizing hormone; E2, estradiol; T, testosterone.

**Figure 5:**
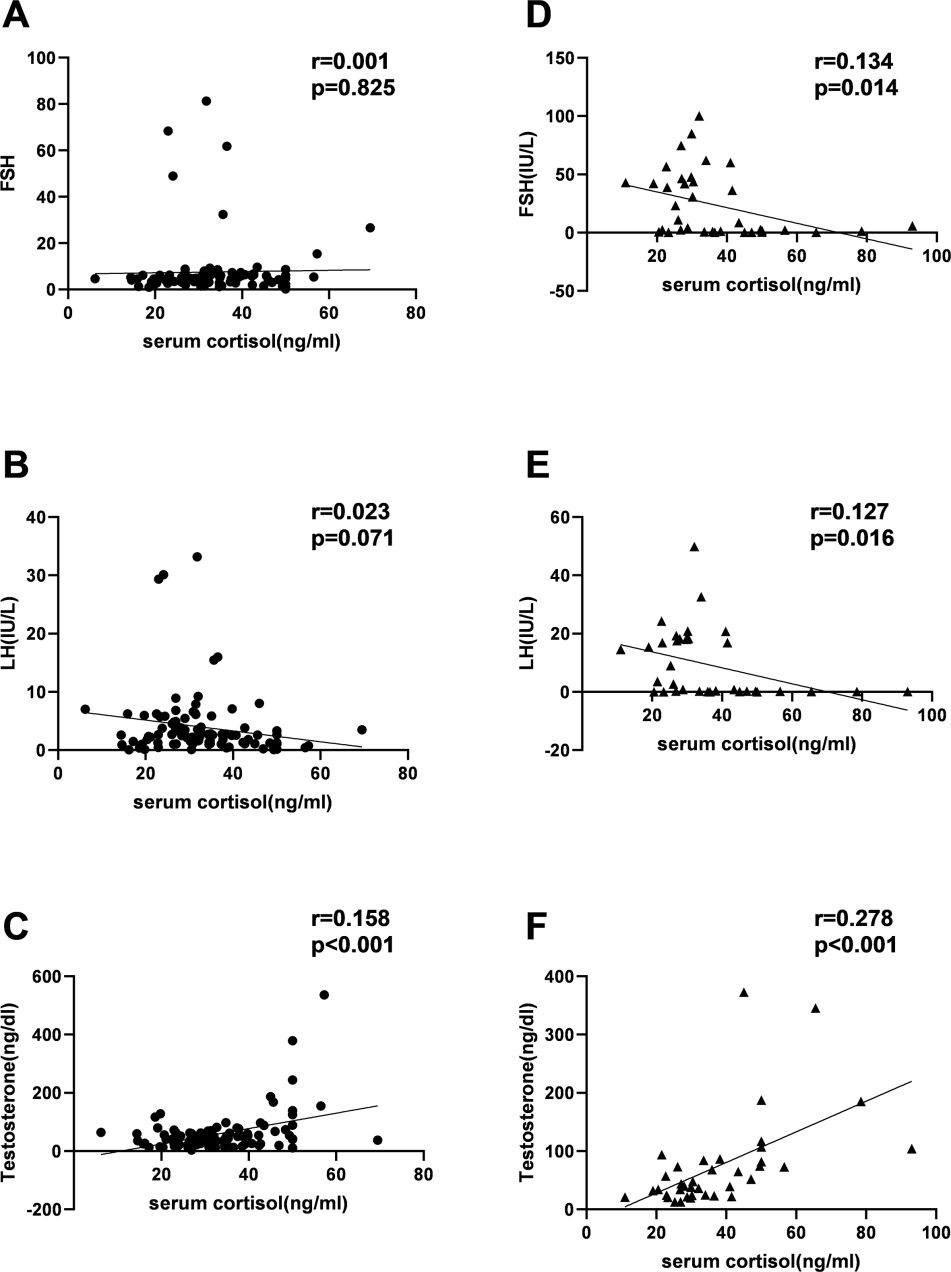
Linear regressions between serum cortisol and reproductive hormones in premenopausal and postmenopausal women. Premenopausal women with Cushing syndrome were included in **(A–C)**. Postmenopausal women with CS were included in **(D–F)** to avoid effects of the menstrual cycle. Pearson correlations(r) were calculated squares. FSH, Follicle-stimulating hormone; LH, Luteinizing hormone.

To investigate the relationship between the severity of hypercortisolism and the HPG axis dysfunction, linear regression analyses were conducted to evaluate the associations between serum cortisol and reproductive hormones. These results were illustrated in **Figure 5** and showed a clear menopausal distinction. In postmenopausal women, serum cortisol exhibited a negative correlation with both FSH (P = 0.014) and LH (P = 0.016), with a positive correlation with testosterone (P < 0.001). In premenopausal women with CS, no significant association was observed between serum cortisol and LH or FSH, although the positive correlation with testosterone persisted (P < 0.001).

Further regression analyses between ACTH, 24h-UFC, and reproductive hormones are presented in **Table 3**. While ACTH closely mirrored serum cortisol, showing notable correlations with LH and FSH only in postmenopausal women, 24h-UFC failed to demonstrate such association, which might be attributed to its known day-to-day and intra-individual variabilit (26). Still, estradiol levels showed no difference across subgroups and did not correlate with any parameters of hypercortisolism, ramaining unaffected by the degree of glucocorticoid excess.

**Table 3:** Correlation between reproductive hormones with 24h-UFC and plasma ACTH levels in premenopausal and postmenopausal women.

Variables	Premenopausal cohort				Postmenopausal cohort			
	24h-UFC		ACTH		24h-UFC		ACTH	
	r	P value	r	P value	r	P value	R	P value
FSH	0.000	0.970	0.003	0.592	0.078	0.049	0.212	0.002
LH	0.000	0.324	0.001	0.276	0.072	0.057	0.212	0.002
TT	0.100	0.001	0.256	<0.001	0.063	0.07	0.197	0.003

Pearson correlations (r) were calculated squares. 24h-UFC, 24h urinary-free cortisol; ACTH, adrenocorticotrophic hormone; FSH, Follicle-stimulating hormone; LH, Luteinizing hormone; TT, total testosterone.

### The characteristics of steroid profiles in adrenal CS and Cushing’s disease

**Figure 6:**
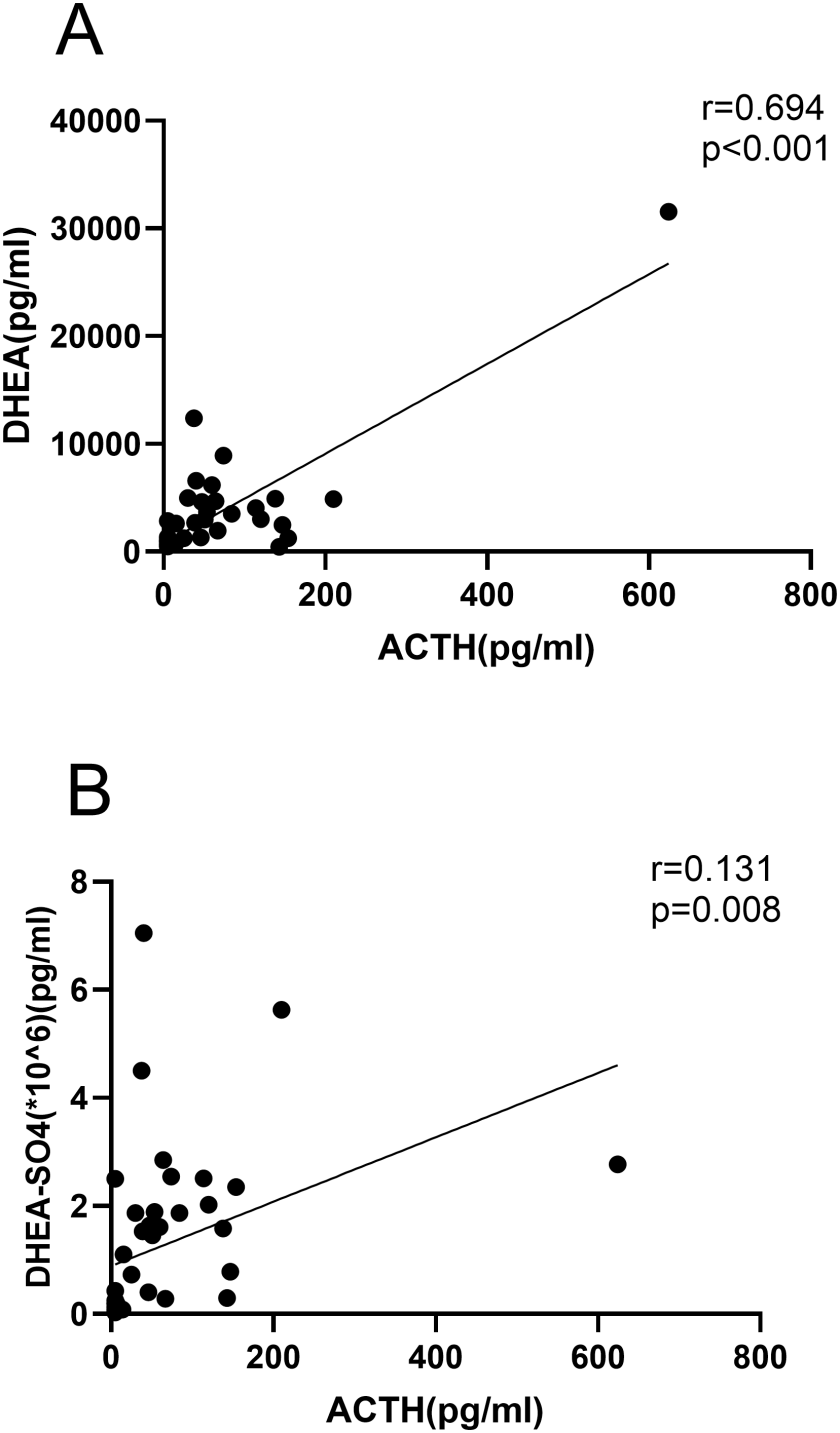
Linear regressions between ACTH and DHEA and DHEAS. The correlations between ACTH and DHEA and DHEAS are displayed in **(A, B)**. Pearson correlations(r) were calculated squares. DHEA, dehydroepiandrosterone; DHEAS, DHEA sulfate; ACTH, adrenocorticotrophic hormone.

The characteristics of steroid profiles were analyzed using MS in 45 women with CS, as detailed in **Table 4**. This subgroup comprised 24 patients with adrenal CS and 21 with CD. Plasma androgens—including testosterone, A2, DHEA, and DHEAS—were significantly elevated in women with CD compared to adrenal CS (P < 0.001) (**Figure 5**). Additional Linear regression analyses between ACTH and DHEA/DHEAS revealed a robust and positive association (**Figure 6**), consistent with the well-established ACTH dependency of adrenal androgen synthesis (27). Beyond the increase in plasma androgens, women with CD also exhibited moderate decreases in 11-deoxycortisol (P = 0.011) and 11-deoxycorticosterone (P = 0.006).

**Table 4:** Characteristics of steroid profile in 45 women with CS.

Variables	Adrenal Cushing syndrome (n=24)	Cushing’s disease (n=21)	P-value
Age (years)	43.75 ± 11.80	39.00 ± 9.84	0.257
Body mass index (kg/m2)	27.47 ± 4.35	26.81 ± 4.84	0.633
Progesterone (pg/ml)	116.00(115.00,217.50)	120.00(100.00,299.50)	0.936
17-hydroxyprogesterone (pg/ml)	287.00(240.50,638.75)	532.00(209.00,815.00)	0.481
Testosterone (pg/ml)	107.50(96.00,162.75)	238.00(177.00,317.50)	**<0.001**
Androstenedione (A2) (pg/ml)	602.00(107.50,812.75)	1349.00(960.00,2419.50)	**<0.001**
DHEA (pg/ml)	648.00(556.50,841.00)	4054.00(2589.00,5588.50)	**<0.001**
DHEA-S (*10^6) (pg/ml)	0.14(0.08,0.25)	1.87(1.50,2.66)	**<0.001**
Cortisol (*10^5) (pg/ml)	1.78 ± 0.78	1.90 ± 0.83	0.538
Corticosterone (pg/ml)	3142.00(2128.25,6479.00)	3125.00(1320.00,5111.50)	0.328
11-Deoxycortisol (pg/ml)	811.50(664.25,1636.00)	609.00(397.00,801.00)	**0.011**
11-Deoxycorticosterone (pg/ml)	106.00(96.00,288.15)	96.000(80.00,96.00)	**0.006**
Cortisone (pg/ml)	20386.13 ± 6388.96	21192.76 ± 8661.66	0.716

Statistically significant results (P<0.05) were highlighted in bold. Comparison between the groups was performed using Student’s t-tests or Wilcoxon test if the distribution of the respective variable was not log-normal in any of the groups. DHEA, dehydroepiandrosterone; DHEAS, DHEA sulfate.

**Figure 7:**
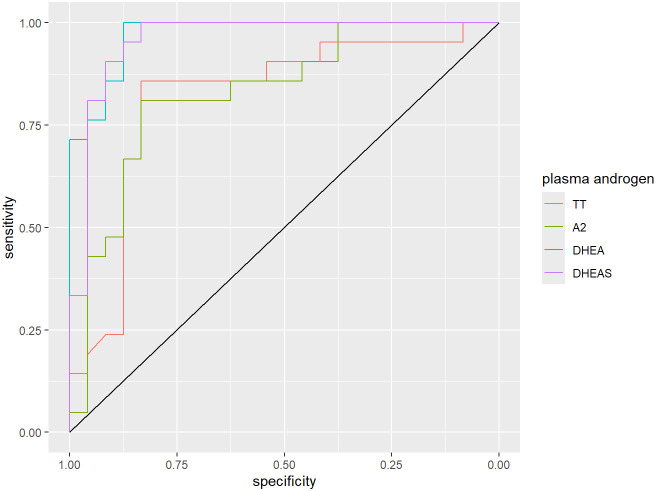
ROC-analysis to differentiate between adrenal CS and CD with plasma androgens. ROC-analysis to differentiate between patients with AI-CS and CD with the most discriminative steroids. TT, total testosterone; A2, androstenedione; DHEA, dehydrospiandrostenedione; DHEAS, dehydrospiandrostenedione sulfate.

To evaluate the diagnostic utility of plasma androgens in distinguishing CD from adrenal Cushing syndrome, ROC analyses were performed, with results detailed in **Table 5** and **Figure 7**. Testosterone demonstrated a sensitivity of 81.0% and a specificity of 83.3%, with a cutoff value of 172.5 pg/ml and an AUC of 0.816. A2 showed a sensitivity of 100.0% and a specificity of 83.3%, with a cutoff value of 917.5 pg/ml and an AUC of 0.829. DHEA exhibited a sensitivity of 100.0% and a specificity of 87.5%, with a cutoff value of 1170.5 pg/ml and an AUC of 0.972. Lastly, DHEA-S had a sensitivity of 100.0% and a specificity of 83.3%, with a cutoff value of 0.27 × 10^6^ pg/ml and an AUC of 0.958. Collectively, these findings indicate that plasma androgens, particularly DHEA and DHEAS, are effective biomarkers for differentiating between adrenal CS and CD, with high sensitivity and specificity at optimal cutoff values.

**Table 5:** Accuracy of steroid biomarkers for differentiating CD form adrenal CS.

Variables	AUC (95% CI)	Sensitivity (%)	Specificity (%)	Youden index
Total Testosterone(TT) > 172.5 pg/ml	0.816 (0.682,0.951)	81.0%	83.3%	0.643
Androstenedione (A2) > 917.5 pg/ml	0.829 (0.706,0.952)	100.0%	83.3%	0.875
DHEA > 1170.5 pg/ml	0.972 (0.934,1.000)	100.0%	87.5%	0.875
DHEA-S > 0.27*10^6 pg/ml	0.958 (0.899,1.000)	100.0%	83.3%	0.833

The AUCs with 95% confidence intervals of the four different androgen were included. The sensitivity and specificity for each marker were derived from the optimal cutoff value. AUC, area under the curve; CI, confidence interval; DHEA, dehydroepiandrosterone; DHEAS, DHEA sulfate.

### Thyroid axis in women patient with Cushing syndrome

**Figure 8:**
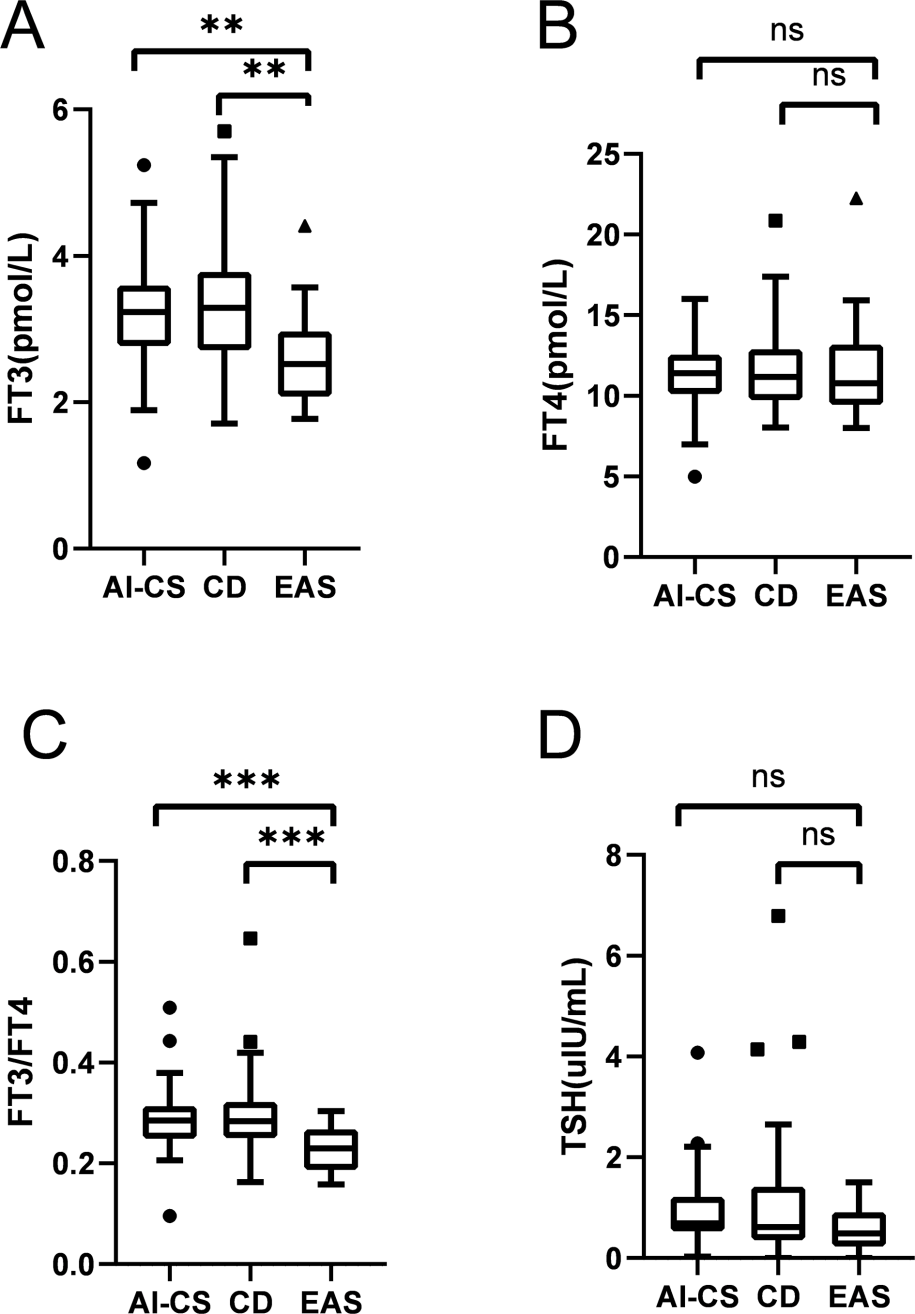
Box-and-whisker plots of thyroid hormone. Serum FT3,FT4,TSH and FT3/FT4 values are displayed in **(A–D)**. All comparison between the three subgroups are shown by brackets,asterisks or text. The observed outlier distribution patterns align with natural biological variation. TSH: thyroid-stimulating hormone; AI-CS, ACTH-independent Cushing syndrome; CD, Cushing’s disease; EAS, ectopic ACTH syndrome. ***P<0.001; **P<0.01; ns, not significant.

Given the interest in the impact of hypercortisolism on the hypothalamic-pituitary-thyroid (HPT) axis, data on thyroid hormone levels were also collected and analyzed, as detailed in **Table 1** and **Figure 8**. As shown, serum free triiodothyronine (FT3) levels in women with EAS were markedly decreased compared to those with adrenal CS and CD (P < 0.01). Neither free thyroxine (FT4) nor TSH levels showed significant differences (**Figures 8B, D**), nor did the three groups after stratification by menopausal status. The FT3:FT4 ratios also showed pronounced distinction in EAS compared to adrenal CS and CD (P<0.001) (**Figure 8C**).

**Figure 9:**
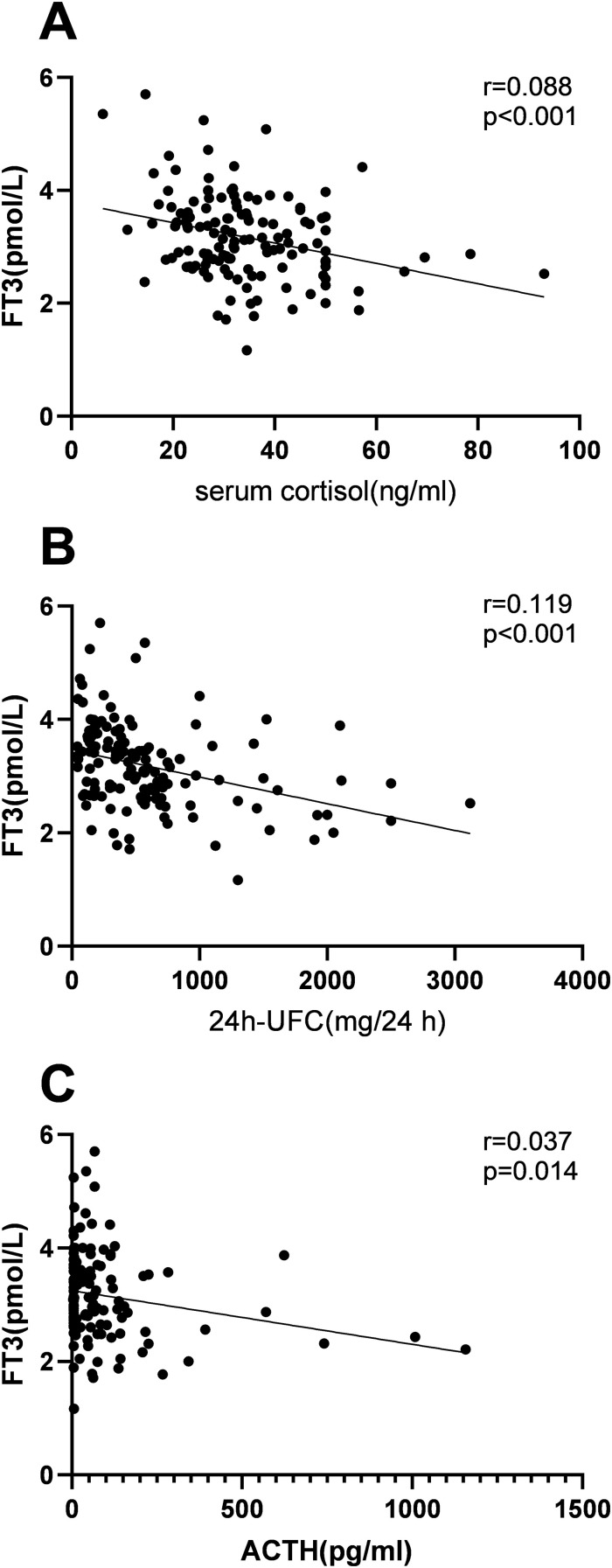
Linear regressions between FT3 and the intensity of hypercortisolism **(A-C)**. Pearson correlations(r) were calculated squares. 24h-UFC, 24h urinary-free cortisol; ACTH, adrenocorticotrophic hormone.

Linear regression analyses were further performed to characterize the relationship between hypercortisolism severity and thyroid function, detailed in **Figure 9**. Among the three parameters, only FT3 correlated with severity of hypercortisolism, showing moderate associations with serum cortisol, 24h-UFC, and ACTH. Neither FT4 nor TSH demonstrated any correlation with the severity of hypercortisolism.

## Discussion

Our study effectively addressed the urgent need to characterize the clinical status of the HPG axis in women with CS of diverse etiologies, demonstrating that hypercortisolism exerts a severity-dependent suppression on the HPG axis. Patients with EAS showed the most pronounced impairment, particularly among postmenopausal women.

The principal finding of this study is the significantly attenuated levels of both LH and FSH in women with EAS, compared to cohorts with adrenal CS and CD (p<0.001). This finding aligns with established evidence demonstrating that EAS typically manifests more severe hypercortisolism relative to other etiologies of Cushing syndrome (2, 5). The marked suppression of LH and FSH provides clinical evidence that hypercortisolism inhibits the HPG axis centrally. This is consistent with the known ability of glucocorticoids to suppress hypothalamic GnRH release and pituitary gonadotropin synthesis (13–15), as well as with other clinical reports of reproductive impairment in women with CS (9, 10). Notably, our study further revealed a pronounced elevation in the FSH: LH ratio in women with EAS. Previous studies have shown that glucocorticoids can differentially regulate FSH and LH secretion at the pituitary level, by potentiating basal FSH while inhibiting LH. The elevated basal FSH: LH ratios, in turn, have been associated with impaired folliculogenesis and diminished oocyte quality (24, 25). Collectively, these findings suggest a dual mechanism contributing to gonadal dysfunction in EAS: (1) glucocorticoid-induced suppression of gonadotropin secretion; (2) dysregulation of follicular maturation dynamics mediated through altered FSH: LH ratios.

Besides the regulation of FSH and LH, we also observed a distinct divergence in circulating androgens among female patients with different CS subtypes. Specifically, female patients with ACTH-dependent CS demonstrated markedly elevated plasma testosterone compared to those with ACTH-independent CS. This finding contrasts with observations in male CS patients, where testosterone levels are suppressed regardless of CS subtype due to direct glucocorticoid-mediated inhibition of testicular Leydig cell steroidogenesis (11, 12, 28). In female, approximately 25% of testosterone is secreted by the adrenal zona fasciculata, another 25% by the ovarian stroma, with the remaining arises from peripheral precursor androgens (29). The elevation of ACTH in ACTH-dependent CS drives a marked increase in adrenal steroid output (30), overriding glucocorticoid-induced suppression of ovarian androgen synthesis, resulting a net increase in circulating testosterone. Consequently, testosterone elevation in women with CS represents a marker of ACTH excess and may offer additional diagnostic utility in discriminating ACTH-dependent from ACTH-independent CS.

No meaningful differences in estradiol levels were observed among the three CS subgroups, nor was there any correlation between estradiol levels and the severity of hypercortisolism, despite the fact that female CS patients are known to have reduced estradiol compared to healthy cohorts (6). A plausible explanation for this intriguing finding may lies in the dual regulatory role of glucocorticoids in ovarian steroidogenesis. Glucocorticoids have been reported to exert both stimulatory and inhibitory effects on ovary. These opposing effects depend on local glucocorticoid concentration, the phase of follicular development, and the differential expression of glucocorticoid receptor isoforms within ovarian cells, leading to differential modulation of progesterone and estradiol production (31). This complexity may explain why clinical reports concerning the effects of glucocorticoids on ovary are scarce and sometimes discordant, suggesting the ovarian effects probably related to the severity of hypercortisolism (32).In our study, the lack of a clear relationship between cortisol excess and estradiol suppression suggested that the stimulatory and inhibitory effects of glucocorticoids on the ovary may coexist and partially offset each other, resulting in the observed stability of estradiol levels across CS subtypes. However, the precise mechanisms underlying these observations remain unclear and warrant further investigation.

To address the lack of menstrual status stratification in prior research (8, 9), we further stratified our study cohort by menopausal status. The results showed that among the three subgroups, postmenopausal women with EAS exhibited the most severe hypercortisolism and HPG axis suppression. Linear regression analyses also demonstrated that the association between serum cortisol and gonadotropins reached statistical significance only in postmenopausal women. This was likely because in premenopausal women, cyclical estrogen and progesterone exert powerful negative feedback on the hypothalamus and pituitary, buffering the suppressive effects of elevated cortisol. In contrast, after menopause, the withdrawal of feedback unmask the effects of glucocorticoids on HPG axis (23). The inverse relationship between serum cortisol and gonadotropins further suggested that hypercortisolism centrally suppresses the HPG axis in a severity-dependent manner, a finding that aligns with previous studies (7–10). However, the correlations were moderate in strength, which was also observed in male populations (11). This finding suggested that hypercortisolism is a major but not exclusive driver of HPG axis suppression. Additional factors, such as individual heterogeneity in GR sensitivity, disease duration, and metabolic comorbidities (31, 33), may also play a modulating role in gonadal impairment.

The steroid profiles of CS have emerged as an important area of research recently, numerous studies have demonstrated that these profiles can aid in diagnosing various subtypes of CS, including clinical and subclinical CS, adrenal CS, CD and EAS (34–36). In this study, the diagnostic potential of plasma androgens in distinguishing CD from adrenal CS was analyzed. The results revealed that all four androgens, including testosterone, A2, DHEA and DHEAS, are effective in differentiating these conditions. Among these, DHEA and DHEAS exhibited the highest sensitivity and specificity, making them valuable supplementary measurements for confirming the causes of CS. Plus, the moderate decreases in 11-deoxycortisol and 11-deoxycorticosterone in women with CD suggested a diversion of precursors toward androgen synthesis (37).

Female CS patients showed selective HPT axis suppression, with decreased FT3 and T3:T4 ratio (both P<0.01) correlating with hypercortisolism severity. This finding was consistent with the known inhibitory effects of hypercortisolism on deiodinase activity, leading to reduced peripheral conversion of T4 to T3 (38, 39). Notably, neither FT4 nor TSH levels showed meaningful differences or correlations with hypercortisolism parameters, as reported in previous studies demonstrating that TSH levels typically do not show a significant reduction (40). Thyroid hormones did not differ significantly among the three CS subtypes in either premenopausal or postmenopausal women, in contrast to marked suppression of LH and FSH. This contrast suggests that pituitary thyrotrophs may be less sensitive to glucocorticoid-mediated inhibition compared to gonadotrophs, and that HPT axis alterations in CS primarily manifest at the peripheral level rather than through central suppression. Given that thyroid hormones are essential for maintaining HPG axis health and can directly influence ovarian function (41, 42), these findings highlight the need for particular attention to HPG axis function in CS patients presenting with concurrent thyroid dysfunction.

The primary limitation of this investigation is its retrospective cross-sectional design, which inherently introduces potential selection bias due to non-randomized participant inclusion and potential information bias from heterogeneous data collection methods. Moreover, this study requires cautious interpretation due to sample size limitations, especially in subgroup analyses for rare etiologies. Female patients with steroid profiles was not stratified by menopausal status cause this subgroups too small for reliable statistical analysis, larger studies are needed to address this gap. In addition, detailed data regarding menstrual cycle phases in premenopausal women were unavailable, potentially masking underlying hormonal associations. Of note, the observed weak correlation between 24h-UFC and reproductive hormones in this study may reflect both methodological constraints and the intra-individual biological variability inherent to 24h-UFC (43), suggesting that single measurements may not adequately capture the chronic hypercortisolism burden.

The principal strength of this study lies in its comprehensive characterization of the clinical spectrum of CS on HPG axis in women(n=137). While the detrimental effects of high-dose glucocorticoids on reproductive function are well-documented, only a limited number of studies have systematically evaluated the clinical manifestations across different CS subtypes (8, 9, 44, 45). This study addresses this critical gap in the literature by employing a stratified analysis based on both etiology and menopausal status, as previous studies have not analyzed premenopausal and postmenopausal women separately, thus provides novel insights into the differential effects of hypercortisolism on reproductive function. An additionally innovative aspect of this study is the incorporation of MS-based steroid profiling, which demonstrated significant utility of plasma androgen in distinguishing CD from adrenal CS. Furthermore, given the frequent impairment of both the HPT and HPG axes in CS (41), our findings underscore the critical need to monitor HPG axis function in CS patients who coexist thyroid dysfunction.

In conclusion, female patients with CS demonstrated central impairment of the HPG axis that correlates with the severity of hypercortisolism,with more pronounced gonadotropin suppression observed in postmenopausal women. Patients with EAS exhibited the most profound suppression of gonadotropin secretion, accompanied by elevated circulating testosterone levels. The steroid profile further provided diagnostic utility for DHEA and DHEAS n discriminating ACTH-dependent from ACTH-independent CS. Additional comparative analysis revealed greater sensitivity of the HPG axis than the HPT axis to glucocorticoid excess. A preprint has previously been published on bioRxiv (46).

## Data Availability

Due to the sensitivity nature of the data, access is restricted by institutional policy and ethical approval requirements. Data are available from the corresponding author upon reasonable request and from permission from the Tianjin Medical University General Hospital.

## References

[1] Reincke M Fleseriu M . Cushing syndrome: a review. JAMA. (2023) 330:170–81. doi: 10.1001/jama.2023.11305 37432427

[2] Gadelha M Fleseriu M Pivonello R Tabarin A Witek P Lima J . Cushing's syndrome. Lancet. (2023) 402(10418):2237–2252. doi: 10.1016/s0140-6736(23)01961-x 37984386

[3] Giuffrida G Ferraù F Lauretta R Baldelli R Appetecchia M Paragliola RM . Global Cushing's disease epidemiology: a systematic review and meta-analysis of observational studies. J Endocrinol Invest. (2022) 45(6):1235–1246. doi: 10.1007/s40618-022-01754-1 35133616

[4] Hayes AR Grossman AB . Distinguishing Cushing's disease from the ectopic ACTH syndrome: needles in a haystack or hiding in plain sight? J Neuroendocrinol. (2022) 34:e13137. doi: 10.1111/jne.13137 35980277 PMC9542389

[5] Ragnarsson O Juhlin CC Torpy DJ Falhammar H . A clinical perspective on ectopic Cushing's syndrome. Trends Endocrinol Metab. (2024) 35(4):347–360. doi: 10.1016/j.tem.2023.12.003 38143211

[6] Pivonello R Isidori AM De Martino MC Newell-Price J Biller BM Colao A . Complications of Cushing's syndrome: state of the art. Lancet Diabetes Endocrinol. (2016) 4(7):611–629. doi: 10.1016/s2213-8587(16)00086-3 27177728

[7] Ferriere A Tabarin A . Cushing's disease. Presse Med. (2021) 50:104091. doi: 10.1016/j.lpm.2021.104091 34718112

[8] Ferrau F Alessi Y Nista F Roux A Ferone D Arvat E . Who and how to screen for endogenous hypercortisolism among young women presenting with clinical hyperandrogenism and/or menstrual abnormalities. J Endocrinol Invest. (2025) 48(Suppl 1):83–89. doi: 10.1007/s40618-025-02537-0 PMC12031912 39982685

[9] Akirov A Dery L Fleseriu M Rudman Y Shimon I Manisterski Y . Cushing's syndrome in women: age-related differences in etiology and clinical picture. Pituitary. (2023) 26(1):144–151. doi: 10.1007/s11102-022-01292-2 36515786

[10] Lado-Abeal J Rodriguez-Arnao J Newell-Price J Perry L Grossman AB Besser GM . Menstrual abnormalities in women with Cushing's disease are correlated with hypercortisolemia rather than raised circulating androgen levels. J Clin Endocrinol Metab. (1998) 83(9):3083–3088. doi: 10.1210/jc.83.9.3083 9745407

[11] Papadakis GE de Kalbermatten B Dormoy A Salenave S Trabado S Vieira-Pinto O . Impact of Cushing's syndrome on the gonadotrope axis and testicular functions in men. Hum Reprod. (2023) 38(12):2350–2361. doi: 10.1093/humrep/dead187 37742130

[12] Zheng H Wang Q Cui Q Sun Q Wu W Ji L . The hypothalamic-pituitary-gonad axis in male Cushing's disease before and after curative surgery. Endocrine. (2022) 77(2):357–362. doi: 10.21203/rs.3.rs-1515304/v1 35639244

[13] Whirledge S Cidlowski JA . Glucocorticoids and reproduction: traffic control on the road to reproduction. Trends Endocrinol Metab. (2017) 28:399–415. doi: 10.1016/j.tem.2017.02.005 28274682 PMC5438761

[14] Joseph DN Whirledge S . Stress and the HPA axis: balancing homeostasis and fertility. Int J Mol Sci. (2017) 18:2224. doi: 10.3390/ijms18102224 29064426 PMC5666903

[15] Huang Y Liu Q Huang G Wen J Chen G . Hypothalamic Kisspeptin neurons regulates energy metabolism and reproduction under chronic stress. Front Endocrinol (Lausanne). (2022) 13:844397. doi: 10.3389/fendo.2022.844397 35685211 PMC9170882

[16] Martinez GJ Appleton M Kipp ZA Loria AS Min B Hinds TD . Glucocorticoids, their uses, sexual dimorphisms, and diseases: new concepts, mechanisms, and discoveries. Physiol Rev. (2024) 104(1):473–532. doi: 10.1152/physrev.00021.2023 PMC11281820 37732829

[17] Mihm M Gangooly S Muttukrishna S . The normal menstrual cycle in women. Anim Reprod Sci. (2011) 124:229–36. doi: 10.1016/j.anireprosci.2010.08.030 20869180

[18] Karavitaki N Ioannidis G Giannakopoulos F Mavrokefalos P Thalassinos N . Evaluation of bone mineral density of the peripheral skeleton in pre- and postmenopausal women with newly diagnosed endogenous Cushing's syndrome. Clin Endocrinol (Oxf). (2004) 60(2):264–270. doi: 10.1111/j.1365-2265.2004.01968.x 14725690

[19] Alhajeri A Hajji S Aljenaee K . Amenorrhea as a presentation of Cushing's syndrome. Endocrinol Diabetes Metab Case Rep. (2024) 2024:e230152. doi: 10.1530/edm-23-0152 39042723 PMC11301536

[20] Nieman LK Biller BM Findling JW Newell-Price J Savage MO Stewart PM . The diagnosis of Cushing's syndrome: an Endocrine Society Clinical Practice Guideline. J Clin Endocrinol Metab. (2008) 93(5):1526–1540. doi: 10.1530/eje-09-0695 PMC2386281 18334580

[21] Görges R Knappe G Gerl H Ventz M Stahl F . Diagnosis of Cushing's syndrome: re-evaluation of midnight plasma cortisol vs urinary free cortisol and low-dose dexamethasone suppression test in a large patient group. J Endocrinol Invest. (1999) 22(4):241–249. doi: 10.1007/BF03343551 10342356

[22] Zhang J Yu H Shen Y Yang X Wang Y . Rapid liquid chromatography-tandem mass spectrometry method for determination of total and free testosterone in human serum and its application to monitoring biomarker response of elite athletes. Molecules. (2024) 29(21):5007. doi: 10.3390/molecules29215007 39519647 PMC11547523

[23] Jabbour HN Kelly RW Fraser HM Critchley HO . Endocrine regulation of menstruation. Endocr Rev. (2006) 27(1):17–46. doi: 10.1210/er.2004-0021 16160098

[24] He Y Liu L Yao F Sun C Meng M Lan Y . Assisted reproductive technology and interactions between serum basal FSH/LH and ovarian sensitivity index. Front Endocrinol (Lausanne). (2023) 14:1086924. doi: 10.3389/fendo.2023.1086924 37206442 PMC10190590

[25] Barroso G Oehninger S Monzó A Kolm P Gibbons WE Muasher SJ . High FSH:LH ratio and low LH levels in basal cycle day 3: impact on follicular development and IVF outcome. J Assist Reprod Genet. (2001) 18(9):499–505. doi: 10.1023/a:1016601110424 PMC3455729 11665665

[26] Wright K van Rossum EFC Zan E Werner N Harris A Feelders RA . Emerging diagnostic methods and imaging modalities in Cushing's syndrome. Front Endocrinol (Lausanne). (2023) 14:1230447. doi: 10.3389/fendo.2023.1230447 37560300 PMC10407789

[27] Vaidya A Findling J Bancos I . Adrenal insufficiency in adults: a review. JAMA. (2025) 334:714–25. doi: 10.1001/jama.2025.5485 40522647

[28] Ren L Zhang Y Xin Y Chen G Sun X Chen Y . Dysfunction in Sertoli cells participates in glucocorticoid-induced impairment of spermatogenesis. Mol Reprod Dev. (2021) 88(6):405–415. doi: 10.1002/mrd.23515 34032349

[29] Burger HG . Androgen production in women. Fertil Steril. (2002) 77:S3–5. doi: 10.1016/s0015-0282(02)02985-0 12007895

[30] Turcu A Smith JM Auchus R Rainey WE . Adrenal androgens and androgen precursors-definition, synthesis, regulation and physiologic actions. Compr Physiol. (2014) 4(4):1369–1381. doi: 10.1002/j.2040-4603.2014.tb00590.x PMC4437668 25428847

[31] Bhaumik S Lockett J Cuffe J Clifton VL . Glucocorticoids and their receptor isoforms: roles in female reproduction, pregnancy, and foetal development. Biology (Basel). (2023) 12(8):1104. doi: 10.3390/biology12081104 37626990 PMC10452123

[32] Castinetti F Brue T . Impact of Cushing's syndrome on fertility and pregnancy. Ann Endocrinol (Paris). (2022) 83:188–90. doi: 10.1016/j.ando.2022.04.001 35443159

[33] Fleseriu M Varlamov EV Hinojosa-Amaya JM Langlois F Melmed S . An individualized approach to the management of Cushing disease. Nat Rev Endocrinol. (2023) 19(10):581–599. doi: 10.1038/s41574-023-00868-7 37537306

[34] Hines JM Bancos I Bancos C Singh RD Avula AV Young WF . High-resolution, accurate-mass (HRAM) mass spectrometry urine steroid profiling in the diagnosis of adrenal disorders. Clin Chem. (2017) 63(12):1824–1835. doi: 10.1373/clinchem.2017.271106 28814383

[35] Braun LT Osswald A Zopp S Rubinstein G Vogel F Riester A . Delineating endogenous Cushing's syndrome by GC-MS urinary steroid metabotyping. EBioMedicine. (2024) 99:104907. doi: 10.1016/j.ebiom.2023.104907 38128413 PMC10776922

[36] Ahn CH Lee C Shim J Kong SH Kim SJ Kim YH . Metabolic changes in serum steroids for diagnosing and subtyping Cushing's syndrome. J Steroid Biochem Mol Biol. (2021) 210:105856. doi: 10.1016/j.jsbmb.2021.105856 33647522

[37] Naamneh Elzenaty R du Toit T Fluck CE . Basics of androgen synthesis and action. Best Pract Res Clin Endocrinol Metab. (2022) 36:101665. doi: 10.1016/j.beem.2022.101665 35595638

[38] Paragliola RM Corsello A Papi G Pontecorvi A Corsello SM. Cushing's syndrome effects on the thyroid. Int J Mol Sci. (2021) 22:3131. doi: 10.3390/ijms22063131 33808529 PMC8003177

[39] Anilkumar AS Thomas SM Veerabathiran R . Cushing's disease and autoimmune thyroid disorders in women: impacts on fertility, pregnancy, and menopause. J Reprod Immunol. (2026) 174:104870. doi: 10.1016/j.jri.2026.104870 41793937

[40] Shekhar S McGlotten R Auh S Rother KI Nieman LK. . The hypothalamic-pituitary-thyroid axis in Cushing syndrome before and after curative surgery. J Clin Endocrinol Metab. (2021) 106:e1316–31. doi: 10.1210/clinem/dgaa858 33236107 PMC7947758

[41] Brown EDL Obeng-Gyasi B Hall JE Shekhar S. The thyroid hormone axis and female reproduction. Int J Mol Sci. (2023) 24:9815. doi: 10.3390/ijms24129815 37372963 PMC10298303

[42] Morenas R Singh D Hellstrom WJG . Thyroid disorders and male sexual dysfunction. Int J Impot Res. (2024) 36:333–8. doi: 10.1038/s41443-023-00768-4 37752332

[43] Petersenn S Newell-Price J Findling JW Gu F Maldonado M Sen K . High variability in baseline urinary free cortisol values in patients with Cushing's disease. Clin Endocrinol (Oxf). (2014) 80:261–9. doi: 10.1111/cen.12259 23746264 PMC4231220

[44] Kaltsas GA Korbonits M Isidori AM Webb JA Trainer PJ Monson JP . How common are polycystic ovaries and the polycystic ovarian syndrome in women with Cushing's syndrome? Clin Endocrinol (Oxf). (2000) 53:493–500. doi: 10.1046/j.1365-2265.2000.01117.x 11012575

[45] Keskin FE Özkaya HM Ortaç M Salabaş E Kadıoğlu A Kadıoğlu P . Sexual function in women with Cushing's syndrome: a controlled study. Turk J Urol. (2018) 44:287–93. doi: 10.5152/tud.2018.74240 29932397 PMC6016657

[46] Yu A Liu X Chen Y Li S Liu M . Impact of Cushing’s syndrome on the hypothalamus-pituitary-gonad axis in women. medRxiv. (2024), 2024.10.30.24316413. doi: 10.1101/2024.10.30.24316413

